# Analysis of Panels of Chemical Biomarkers in the Honeybee in Hemolymph and Fat Body in Response to Physiological and Environmental Factors

**DOI:** 10.3390/metabo15110743

**Published:** 2025-11-16

**Authors:** Maciej Sylwester Bryś

**Affiliations:** Department of Invertebrate Ecophysiology and Experimental Biology, University of Life Sciences in Lublin, Doświadczalna 50a, 20-280 Lublin, Poland; maciej.brys@up.lublin.pl

**Keywords:** biomarkers, metabolites, ecophysiology, stressors, insects, *Apis mellifera*

## Abstract

This review synthesizes current knowledge on chemical biomarker panels in the honeybee in a tissue-specific and factor-oriented framework. We show that these panels undergo predictable shifts under endogenous factors (age, caste) and environmental stressors, including mites, bacteria, fungi, viruses, pesticides, antibiotics, adulterated wax, nutritional deficits, and monodiets. These changes are particularly evident in the hemolymph and fat body and are assessed via markers of energy metabolism, enzymatic activities, oxidative stress, and lipid homeostasis. Because insects lack established clinical reference intervals, emphasis is placed on general trends and tissue interrelationships. Moreover, in the honeybee, patterns can at times be inverted relative to vertebrates for example, for enzymatic activities marker, where increased activity may indicate a beneficial effect on the organism. Research in bee ecophysiology is gaining prominence and aligns with contemporary understandings of global challenges.

## 1. Introduction

The honeybee is a eusocial organism, meaning it has reached the highest level of sociality among animals [1]. Colony immunity is determined through the hygienic behavior of worker bees as well as bee products [2]. The maintenance of colony homeostasis is ensured by individual organisms. With age, in particular castes, especially in queens and workers, changes are observed at the anatomical, physiological, biochemical, and molecular levels [3,4]. In addition to endogenous factors, the physiology of individuals is affected by stress-inducing factors such as *Varroa destructor* [5], *Variomorpha* spp./*Nosema* spp. [6] *Paenibacillus larvae* [7], *Ascosphaera apis* [8], deformed wing virus (DWV) [9], pesticides [10,11,12,13,14,15,16], antibiotics [17], adulterations of beeswax [18,19], climate warming [20], nutritional deficiencies and monodiets [19,21,22,23], as well as combined stress [24,25,26]. Exposure to the above-mentioned factors leads to disturbances of metabolic homeostasis. These changes are particularly observed in the hemolymph, the fat body, and other tissues. Understanding the relationships between these tissues provides valuable information on the organism’s response to stress and indicates premature aging-related changes [27,28].

Honeybees, due to their well-characterized genome, ease of rearing, small size, and rapid reproductive processes, are excellent model organisms [29]. In the context of biomedical research on *A. mellifera*, the obtained results on biochemical parameters as well as mechanisms of learning, encoding, and memory are similar or analogous to those in humans [30]. This universal applicability of bees as research models means that the methods and terminology used in medicine have also found application in entomology. The term “biological marker” or “biomarker” originates in the medical field and is defined as a characteristic objectively measured and evaluated as an indicator of biological processes, pathogenic processes, or as an indicator of the action of xenobiotics [31]. Biomarkers are defined as measurable variations at the molecular, cellular, physiological, and behavioral levels, which manifest primarily under the influence of substances acting on the organism [32].

According to their intended purpose, biomarkers should be characterized by sensitivity, specificity, reproducibility, and stability, with ease of diagnosis a desirable feature [32]. The analysis of metabolomic processes has significantly contributed to the improvement of assessment methods, for example, in evaluating the effects of biostimulators in the honeybee [33,34,35,36]. Current discoveries of biomarkers in insects and larvae are used to assess the influence of environmental stressors, particularly in monitoring xenobiotic metabolites [37,38,39].

The interactions between diet composition and the functioning of the honeybee organism indicate complex relationships involving the nutritional value of food, physiological pathways, and the condition of the entire colony. The effect of diet on gene expression constitutes an important, yet separate area of research that goes beyond the scope of this review and requires dedicated discussion.

In honeybees, both individual and social immunity mechanisms are distinguished. In addition to anatomical-physiological barriers (digestive system, respiratory system, and cuticle), individual immunity includes cellular immunity and humoral immunity, which consists of biochemical immunity. The latter is composed of compounds of the proteolytic system, the antioxidant system, and biochemical markers; immune proteins in hemolymph, e.g., phenoloxidase, lysozyme, as well as other immune proteins such as melittin and royalisin [40,41,42,43,44]. Mechanisms of cellular and biochemical immunity are activated simultaneously after pathogens breach the anatomical and physiological barriers. Compounds of the proteolytic system “cut” specific pathogen proteins into small units. The products of these reactions, along with reactive oxygen species (ROS) and toxic metabolites, are neutralized and detoxified by the antioxidant system. Biochemical markers complement these two systems and are considered indicators of honeybee health [45,46]. However, the interpretation of metabolic trends based on single-factor studies, as summarized in the comparative tables presented in this review, may not fully reflect the complexity of biochemical responses in honeybees. Therefore, it is essential to further develop biochemical analyses using hemolymph or tissue-specific samples, which can provide a more precise characterization of metabolic pathways. Traditionally, most assessments of honeybee biochemistry have relied on homogenates of whole abdomens [47,48,49,50,51], which may mask tissue-specific variations in enzymatic activity and metabolite distribution. Expanding studies toward organ or fluid-based analyses will enable a more accurate understanding of the physiological mechanisms underlying responses to environmental factors.

The aim of this review is a comprehensive analysis of the literature and the collection of current and key data on panels of biochemical markers in the honeybee in hemolymph and fat body in response to physiological and environmental factors.

## 2. Energetic Metabolism Biomarkers

Carbohydrate metabolism plays a key role in the physiology of the honeybee, determining their vital activity, immunity, and ability to survive unfavorable environmental conditions (rainy season, starvation), as well as ensuring proper overwintering. Nectar constitutes the main source of sugars in the bee diet, whereas pollen provides primarily proteins, amino acids, and other nutrients [52]. However, filling the gaps in knowledge about bee nutrition reveals that it is not only proteins but also carbohydrates that form the foundation of the biochemical processes of the honeybee [53]. Simple sugars represent the direct metabolic fuel for intensive life processes such as flight, thermoregulation, and royal jelly secretion. During periods of interruption in access to natural forage, both in regions with prolonged rainy seasons and during weakened colony development, it becomes necessary to supplement the diet through carbohydrate feeding [53,54].

There is no doubt that carbohydrate nutrition forms the basis for maintaining homeostasis, especially in the context of immunity against stress-inducing factors. Table 1 presents the general tendencies of glucose concentration across different tissues under the influence of various factors. The most commonly used carbohydrate foods by beekeepers are sucrose syrup, corn syrup, and inverted sugar [54]. Fructose and glucose are simple sugars, referred to as “energy fuels,” which are rapidly metabolized and immediately provide energy. In the case of inverted sugar, being a mixture of glucose and fructose, as well as in corn syrup, no additional enzymatic breakdown is required, which accelerates their utilization. However, the most frequently used energy source remains sucrose syrup prepared from beet sugar, as well as commercially available “sugar candy,” containing sucrose as the main component. Sucrose, being a disaccharide, requires enzymatic hydrolysis by invertase secreted by bees, and the products of this process are primarily glucose and fructose.

Already in the twentieth century, it was demonstrated that feeding bees sucrose syrup extended their survival under laboratory conditions but did not cause significant changes in physiological parameters [55,56]. In light of more recent analyses, the mere extension of lifespan cannot be regarded as the sole indicator of metabolic success; it becomes necessary to adopt a holistic approach that considers immunity, the ability to survive at low temperatures, and the colony’s ability to maintain metabolic balance [53].

The type of sugar provided determines not only the rate of energy release but also the profile of metabolites present in the hemolymph. Regardless of whether the carbohydrate source is a disaccharide or a polysaccharide, the main metabolic products are monosaccharides (glucose or fructose) and trehalose, the principal circulating sugar in the hemolymph. It is glucose and trehalose that are recognized as energy biomarkers of bees, whose levels in hemolymph reflect both the current metabolic rate and the condition of the colony. There is a strong quantitative relationship between the glucose content and trehalose content in the hemolymph, based on a study by Arslan et al. [57], whereas some more recent studies indicate that the relationship between sugar levels may be more complex [58].

Moreover, the type and quality of sugars available in the natural environment largely determine colony functioning. In environments dominated by monocultures, a short-term oversupply of carbohydrates is observed, followed by a period of deficit, which requires support from the beekeeper [59]. In diverse landscapes, rich in melliferous plant species with different phenologies, access to sugars is more evenly distributed, which promotes metabolic stability and bee immunity.

In light of the above data, it is clear that regardless of the source, natural or artificial, the end products of metabolism are mainly glucose and trehalose, which determine the dynamics of physiological processes. However, it is environmental conditions, plant phenology, and beekeeping practices that dictate the availability and quality of carbohydrates, and thus the stability of the entire colony. Understanding these interrelationships is of fundamental importance both in ecophysiological research and in apicultural practice [60].

**Table 1:** General tendencies of glucose concentrations in different tissues of honeybees under the influence of various factors.

Factor	Tissue	Trend	Literature
**Age**	hemolymph	↑↓	[22,61] [34]
**Flight/muscle activity**	thoracic muscle	↑	[62]
	hemolymph	↓	[63]
**Food deprivation**	hemolymph	↓	[64]
**E-field at frequency 50 Hz**	hemolymph	↓	[65]
**Deformed wing virus infection**	hemolymph	↑	[66]
***Nosema* spp.**	hemolymph	↓↑	[6] [67]
***Varroa destructor***	hemolymph	↓	[68]
**CBD extract**	hemolymph	↑	[34]
**Neonicotinoid pesticides**	hemolymph	↓	[69]
**Exposure to common tansy extract (a natural substitute for synthetic pesticides)**	hemolymph	↑	[70]
**Fungicide (active ingredients: boscalid and pyraclostrobin)**	hemolymph	↑	[71]
**Formamidine amitraz**	hemolymph	↑	[72]
**Treatment with bromfenvinphos**	hemolymph	↓	[73]
**Treatment with amphotericin-B**	hemolymph	~	[17]
**Consumed curcumin**	hemolymph	↑	[35]
**Various monofloral and polyfloral diets**	hemolymph	↑	[22,74]
	fat body	↑	[22]

↑ increase, ↓ decrease, ~ not much difference.

## 3. Total Protein as a Biomarker

In human and veterinary medicine, serum proteins are valuable indicators allowing for the assessment of health conditions and nutritional status of the organism. Similarly, in honeybees, total protein is one of the key nutrients, and its main source remains floral pollen [75,76]. The content of total protein in dry matter may range from 2.5% up to even 61% [77]. For a long time, the nutritional value of pollen was assessed primarily on the basis of its total protein content, treating this parameter as the fundamental determinant of food quality [78]. However, filling the gaps in knowledge about the biological value of pollen has revealed that such a perspective is overly simplified; it is not the protein level itself, but rather its amino acid composition, especially the share of essential amino acids, that determines the actual nutritional value [23,79]. Equally important is the presence of essential saturated and unsaturated fatty acids, which influence the energy and immune metabolism of bees [80]. Completing this assessment is the profile of macro and microelements, including stoichiometric ratios (e.g., Ca, P, K, Na), which determine both the bioavailability of minerals and the efficiency of metabolic processes [81]. A properly balanced diet may improve survival by shaping resistance to parasites, viruses, bacteria, and fungi [82,83].

In the context of nutritional assessment, the total protein concentration in hemolymph or tissues constitutes a reliable indicator, since it correlates with diet quality [78]. Moreover, vitellogenin, being one of the major proteins of hemolymph in adult bees, is also a useful parameter for nutritional assessment, due to its dependence on both the quality and quantity of ingested food [84,85]. Moreover, vitellogenin is also closely linked to the immune capacity of both workers and queens. Nicewicz et al. [39] proposed that vitellogenin levels may serve as a biomarker of colony strength, with the potential to predict colony fate.

Hexamerins are storage proteins that play a key role as a source of amino acids. Their concentration changes dynamically during bee development. It is particularly high during metamorphosis, when the organism utilizes its reserves for intensive remodeling processes. After the emergence of adult individuals, the concentration of hexamerins decreases significantly, which indicates the termination of their storage function [86]. Major royal jelly proteins also play an important role in metamorphosis, providing larvae with essential amino acids and supporting resistance against pathogens such as *Paenibacillus larvae* [87]. Ferritin is also present in bee hemolymph, occurring in two isoforms: the heavy chain (type H) and the light chain (type L). Its concentrations increase after emergence, which may be related to the preparation of the immune system to cope with stress and environmental challenges after leaving the brood cell. As in other organisms, ferritin functions as an immune regulator, protecting against excessive immune response and acting as a protective element against oxidative stress [88]. In honeybees, transferrin *Tsf1* plays a role in iron transport in hemolymph. Iron is an essential element for many oxidoreductive enzymes and metabolic processes, yet in its free form, it is toxic. Its binding by transferrin protects cells against oxidative stress. Moreover, transferrin exhibits additional immune functions: it limits the availability of iron to pathogens, so-called ‘nutritional immunity’, which hinders the development of microorganisms such as *Nosema* spp. or opportunistic bacteria. It has been shown that the expression of *AmTsf1* increases under the influence of environmental stressors and infections, which suggests its importance in the immune response and in maintaining iron homeostasis [89].

Heat shock proteins (HSPs) were also detected in the hemolymph of bees in the pupal stage; however, their amount was significantly lower than in the prepupal stage. HSPs are synthesized both constitutively and induced by environmental stressors such as elevated temperature, and play an important role in metamorphosis processes. Higher concentrations of HSPs in pupal hemolymph reflect the increased demand for protein synthesis at this stage. Other proteins, including metabolic and energetic ones, also support the intensive processes of organogenesis, such as the development of the brain, hypopharyngeal glands, and the olfactory system. An important group consists of cytoskeletal proteins (e.g., actin, tubulin, myosin), which are responsible for cell shape, division, and differentiation, as well as odorant-binding proteins related to chemical communication and spatial orientation [84].

The botanical origin of pollen determines both the amount and the quality of protein, and thus the availability of essential amino acids. Bees exhibit high efficiency in digesting pollen; this process reaches about 75% efficiency, whereas the digestibility of proteins from alternative sources, such as soybean or yeast preparations, does not exceed 25% [85]. For this reason, natural pollen constitutes an irreplaceable source of protein, and the level of total protein in the bee organism is directly dependent on the quality and availability of this food. Moreover, protein concentration in hemolymph and fat body changes dynamically depending on age and bee role. In nurse bees, protein levels in hemolymph reach the highest values (approx. 4.3%), which is related to the intensive synthesis of hypopharyngeal gland secretions, whereas in foragers, it decreases to approx. 1.7%, since their metabolism shifts towards intensive utilization of carbohydrates as an energy source [86]. This variability also applies to the annual cycle in April, the protein concentration in hemolymph averaged 13 mg/mL, whereas in November it increased to 54.4 mg/mL [87].

The second key center of protein metabolism in bees is the fat body, functioning analogously to the liver and adipose tissue of vertebrates [22,46]. This structure accumulates proteins, lipids, and glycogen, serving as a metabolic reservoir. In the larval stage, it contains significant amounts of protein, which are then used during metamorphosis for the construction of adult insect tissues. After pupation, the proteins of the fat body serve as a metabolic reservoir for worker bees [90,91]. During adult life, distinct differences are observed: nurse bees have higher protein levels, enabling royal jelly synthesis, whereas in foragers, the protein content is reduced [92,93]. A special role is played by winter bees, whose fat body is characterized by the highest protein content, ensuring survival during the overwintering period and enabling the feeding of the first spring brood [22,91,94]. Importantly, protein concentrations differ between fat body segments, particularly high values were observed in tergite 5 [22]. A relationship has also been shown between the length and width of trophocytes and protein concentration, including immune, enzymatic, and structural proteins [95,96,97].

The significance of a protein diet goes beyond development and metabolism—it also concerns immunity and defense functions. Compounds derived from pollen (proteins and amino acids) are absorbed from the intestine and transported by hemolymph to the fat body, where the synthesis of most immune proteins occurs [91]. An example is vitellogenin, produced by trophocytes, which possesses strong antimicrobial and anti-aging properties [39]. De Grandi-Hoffman et al. [98] demonstrated that mixtures of spring and autumn pollens have similar total protein concentrations but differ in amino acid composition. Pollens of spring plants, such as rapeseed, contain high concentrations of tryptophan, valine, glutamine, and serine—amino acids that are components of apisimin, a peptide supporting royal jelly synthesis [99]. In turn, autumn pollens are rich in proline and hydroxyproline, supporting thermoregulation and winter survival.

Bees fed with bee bread achieve higher hemolymph protein concentrations and greater survival after *Nosema ceranae*/*Vairimorpha* ceranae infection compared to bees fed with protein substitutes. However, parasitism by *Varroa* spp. led to the proliferation of viral populations and a decline in metabolic activity, particularly through the suppression of protein metabolism [100]. This detrimental effect was not reversed by pollen consumption. This confirms the strong influence of dietary protein on the regulation of immune functions [101]. Moreover, a pollen-rich diet increases the size of venom glands and enhances venom synthesis, whose main components, melittin, apamin, and phospholipase, are of protein and peptide nature [102]. Protein concentration increases with the age of workers; the lowest is observed in one-day-old bees, whose hemolymph has low protein content, likely due to metabolic expenditure associated with histogenesis [88]. Thus, the first feeding represents a critical moment, enabling the synthesis of new proteins and the onset of full metabolic activity.

In a broader environmental context, protein and lipid levels in hemolymph may serve as biomarkers of landscape quality and land use. It has been shown that the transition from short-lived summer bees to long-lived winter bees is accompanied by a significant increase in hemolymph proteins and lipids, including vitellogenin, which allows winter bees to survive for several months relying solely on carbohydrate stores [103]. Therefore, protein in honeybees is not only a metabolic component but also an element strictly connected with the ecology and environmental conditions in which the colony functions (Table 2).

## 4. Enzymatic Biomarkers

Enzyme activities in hemolymph constitute an important indicator of physiological condition and the response to environmental factors. High activities of enzymatic biomarkers in the hemolymph of bees, as well as in the fat body, are interpreted as a marker of good insect condition and properly functioning detoxification mechanisms [110,111]. Transaminase enzymes are synthesized in the fat body and subsequently released into the hemolymph. The group of transaminases includes two enzymes: aspartate transaminase (AST) and alanine transaminase (ALT). Their activities are related both to protein and carbohydrate metabolism. As metabolites, i.e., enzymes catalyzing the transfer of an amino group between amino acids and α-keto acids, they are useful biomarkers in monitoring detoxification metabolism [110,112].

In honeybee hemolymph, elevated levels of AST and ALT, unlike in vertebrates, represent a positive phenomenon indicating good insect condition and modulation of the immune response. An increase in the activities of these enzymes is observed in response to the action of biostimulators such as CBD extract, piperine, caffeine, coenzyme Q10, or curcumin [35,36,102,113,114].

Alkaline phosphatase (ALP) belongs to the hydrolase group and serves as an enzymatic biomarker associated with the transport of nutrients between the midgut, hemolymph, and fat body. In bees, its activity reflects the efficiency of metabolic processes and the physiological state of the insect [110].

Gamma-glutamyl transpeptidase (GGTP) in insects plays a role in the transport of amino acids across cell membranes and participates in glutathione metabolism. Thus, this enzyme is associated with detoxification mechanisms and protection against free radicals. GGTP activity in honeybee hemolymph may increase in response to stress factors, e.g., high temperature and pesticides [115].

Combining analysis of enzyme activity in hemolymph with that in the fat body enables the avoidance of inter-individual differences and minimizes the risk of error. Moreover, this approach reflects the condition of the entire organism (Table 3, Table 4 and Table 5).

## 5. Antioxidant Biomarkers

Oxidative stress is defined as a state of disturbed balance between the production of reactive oxygen species (ROS) and the ability of the organism to neutralize them by means of antioxidant mechanisms. ROS, such as superoxide anion radical (O_2_•^−^), hydroxyl radical (HO•), singlet oxygen (^1^O_2_), and hydrogen peroxide (H_2_O_2_), arise as a result of natural metabolic processes, and their production may be intensified under the influence of various stressors, biotic, abiotic, and anthropogenic [118,119]. At physiological concentrations, ROS play an important role in regulating cellular processes such as differentiation, proliferation, apoptosis, and immune response. However, in excess, they act harmfully and initiate destructive processes leading to lipid peroxidation and damage to cell membranes, mutations in DNA, as well as degradation or improper folding of proteins, which results in disturbances in the functioning of cells, tissues, and entire insect organisms [120].

To counteract the harmful effects of ROS, insects possess a complex antioxidant system. It consists of both enzymatic antioxidants, such as superoxide dismutase (SOD), catalase (CAT), glutathione peroxidase (GPx), and glutathione S-transferase (GST), as well as non-enzymatic antioxidants, which include vitamin C (ascorbic acid), vitamin E (α-tocopherol), coenzyme Q10 (ubiquinone), glutathione (GSH), uric acid, and plant-derived compounds present in the insect diet—carotenoids and flavonoids [121,122]. The general ability of the organism to neutralize reactive oxygen species is determined by the indicator TAC (Total Antioxidant Capacity), which encompasses the combined activities of enzymatic antioxidants and the concentrations of non-enzymatic antioxidants in tissues.

In honeybees, the antioxidant system is conditioned by many factors, such as caste, age, physiological state, or pressure from anthropogenic factors [48]. The activities of antioxidant enzymes (SOD, CAT, GST, GPx) and total antioxidant capacity (TAC) are commonly used as biomarkers of oxidative stress (Table 6). In research practice, determinations are most often carried out in homogenates of whole abdomens [48,49] or in hemolymph [123,124,125,126,127]. Much less attention, however, is devoted to analyses conducted in the fat body, which serves as a key metabolic organ and may represent an important source of knowledge about the oxidative balance of insects.

In the last decade, an increased interest has been observed in natural bioactive substances, mainly of plant origin, which may act as biostimulators. Their effect consists of stimulating the insect immune system by enhancing the activity of both the proteolytic system and the antioxidant system [33,35,125]. Changes in the activities of antioxidant enzymes and levels of TAC are interpreted as the organism’s response to the presence of stress-inducing or stimulating factors. An increase in activity may indicate the mobilization of the insect’s defense system to neutralize excessively produced ROS, whereas a decrease in activity indicates a weakening of the antioxidant barrier and increased susceptibility of the organism to oxidative stress.

## 6. Lipid Metabolic Biomarkers

Lipids play an essential role both in the cell wall of pollen grains, providing protection against desiccation and damage, and enabling adhesion during transport by pollinators and within the cytoplasm, where they serve as nutrients [131]. The primary source of lipids for the honeybee is pollen loads [85,132,133]. The percentage content of lipids depends on botanical origin and ranges from 0.94 to 24.6% [134,135,136].

Lipids are absorbed in the midgut and enter the hemolymph, after which they are stored in fat body cells [87,137,138]. Lipid metabolism in insects is similar to that of vertebrates but less complex. Lipids perform numerous metabolic and structural functions in insects, and their diversity stems from chemical structure. In general, they are divided into simple lipids, complex lipids, and isoprenoid lipids. Simple lipids include glycerides, i.e., esters of glycerol and long-chain fatty acids, among which monoglycerides (MAGs), diglycerides (DAGs), and triacylglycerols (TAGs) are distinguished [139,140]. MAGs are mainly intermediates in fat digestion and metabolism. DAGs play an important role as the primary form of lipid transport in the hemolymph to the trophocytes of the fat body. TAG, in turn, is the principal energy storage form in the bee’s fat body and constitutes the main source of energetic substrates. TAG concentrations, together with glucose, reflect the balance between nutrient intake, storage, and mobilization, and the organism’s energy demand; their disturbances are associated with intensified energetic and oxidative stress. Moreover, 90% of lipids occur as triacylglycerols, which are synthesized from carbohydrates [141]. In view of this, triacylglycerols are used as a biomarker of honeybee energy metabolism both in the hemolymph and in the fat body. The entire process is tightly regulated hormonally by adipokinetic hormone and the neurohormone octopamine. The dynamics of lipogenesis and lipolysis change with developmental stage and environmental conditions. Diverse environmental factors, as well as internal metabolic disturbances, can lead to damage to biological membranes [139]. This process can be monitored by lipid peroxidation, a free radical reaction leading to the oxidation of polyunsaturated fatty acids of cell membranes. As a result, reactive aldehydes are formed, including MDA, which themselves constitute biomarkers of oxidative stress and lipid damage [120,142].

Among complex lipids are phospholipids and glycolipids. Phospholipids are the fundamental components of biological membranes, determining their fluidity and selective permeability. In addition, phospholipids participate in cell signaling pathways. Glycolipids, in turn, occur mainly in cell membranes [142].

In lipid homeostasis, especially in adult bees, the *corpus adiposum* plays a key role. The fat body lies beneath the cuticle and viscerally around the abdominal cavity [95]. The tergital part of the fat body is segmental; morphological differences are observed between segments, including between tergites 3 and 5 and the sternite [95]. The tissue consists mainly of trophocytes and oenocytes. Trophocytes are numerous, large, polygonal/oval cells containing glycogen granules, lipid droplets, and protein structures; they may accumulate uric acid crystals. They are responsible not only for energy storage but also for the synthesis of proteins (e.g., vitellogenin), lipids, and hormones [88]. Their size, shape, and coloration change depending on age, physiological state, and environmental factors. The second cell type is oenocytes (oval, triangular, or spindle-shaped, with a central nucleus), which participate in the synthesis of sex pheromones, lipids, and lipoproteins, as well as in xenobiotic detoxification [143,144]. Functionally, the fat body acts as a “liver, pancreas, spleen, and adipose tissues in vertebrates”; it stores glycogen and TAG, synthesizes proteins, participates in protein turnover and nitrogen metabolism, and in detoxification, and transmits endocrine signals [94]. It is also a key element of honeybee immunity.

The composition and properties of lipids change with age and caste [141]. The unsaturation of membrane phospholipids differs between castes and with age, which may result from differences in lipid intake. Winter bees are characterized by the greatest fat body mass, reflecting the high energy demand for heat production in the winter cluster with limited flight activity [141]. This enlargement is associated with an increase in cell size rather than number. This results from the fact that fat body cells are polyploid and undergo cycles of DNA endoreduplication rather than cell division [94].

Triacylglycerol concentration in honeybee hemolymph undergoes significant fluctuations under stressors (Table 7). Such changes were observed, among others, during parasitic infection caused by *Varroa destructor*, which led to reduced triacylglycerol concentration in the hemolymph, associated with increased energy demand and disruption of metabolic homeostasis [105]. Similar effects were noted during the use of antifungal drugs such as amphotericin B, which led to a decrease in hemolymph triglycerides [17]. In addition, environmental factors such as limited food availability, glucose deficiency, or oxidative stress cause mobilization of triacylglycerol reserves stored in the fat body, leading to their degradation and use as an energy source [142,145].

In light of the above, triacylglycerols are regarded as a valuable biomarker of the energetic state of the honeybee organism. Their concentration reflects metabolic balance and influences immunity, thermoregulation, and the longevity of bees. Therefore, monitoring triglyceride levels in the hemolymph and tissues of bees constitutes an important tool for assessing their physiological status and the effectiveness of nutritional or therapeutic interventions.

## 7. Conclusions and Further Research Directions

The metabolism of honeybees undergoes substantial changes under the influence of both internal factors, such as age and caste, and environmental stressors. The transition of workers from nurses to foragers is associated with a shift in dietary preferences, chiefly an increased demand for carbohydrates and a decreased demand for protein. The change in diet determines physiological changes, e.g., atrophy of the hypopharyngeal glands, ovarian reduction, decreased fat body mass, and an intensification of glycogenolysis and methylation processes [146]. Often exacerbated by stressors, these lead to metabolic dysregulation, impaired immunity, and premature aging [145,147,148,149,150]. Monitoring of these metabolic changes is possible through biochemical biomarkers, which reflect the physiological state of bees and their responses to various environmental and dietary factors. Metabolomic analysis has significantly improved methods for assessing the effects of factors such as biostimulants on bees, and contemporary biomarker studies in insects and larvae enable the assessment of the impact of xenobiotics and other environmental stressors. Worth noting are biomarker panels related to the fat body, as changes in triacylglycerol, protein, and glycogen concentrations can serve as early warning indicators of colony overwintering preparedness.

In the context of the loss of biodiversity of pollen- and nectar-bearing plants, the expansion of invasive species, the disappearance of field margins, the use of herbicides, frequent lawn mowing, and large-scale farming that enforces monodiets, research in the nutritional physiology of bees is gaining major importance and aligns with current scientific discourse.

Promising future research directions still include (1) metabolic interactions under cumulative stressors, (2) links between habitat transformation and physiological–biochemical changes in insects, (3) elucidation of the effects of invasive-plant pollen on pollinator physiology, (4) an interdisciplinary perspective on the plant-pollinator relationship, including the use of technological advances based on artificial intelligence and satellite data, and (5) the development of tests that enable rapid detection of xenobiotics. Understanding these multifaceted relationships will allow for better management of honeybee colonies.

## Figures and Tables

**Table 2:** General tendencies of protein concentrations in different tissues of honeybees under the influence of various factors.

Factor	Tissue	Trend	Literature
**Age**	hemolymph	↑ until a specific time	[35,104]
**Casta (foragers vs. nurses)**	brain tissue	*	[105]
**E-field at frequency 50 Hz**	hemolymph	↑	[65]
**E-field at frequency 900 MHz**	hemolymph	↓	[106]
**CBD extract**	hemolymph	↑	[34]
***Varroa destructor***	hemolymph	↑	[107]
**Treatment with bromfenvinphos**	hemolymph	↓	[73]
**Treatment with amphotericin-B**	hemolymph	↑	[17]
	on the body surface	↑	[108]
**Consumed curcumin**	hemolymph	↑	[35]
**Consumed caffeine**	hemolymph	↑	[36]
**Consumed coenzyme Q10**	hemolymph	↑	[109]
**Various monofloral and polyfloral diets**	hemolymph	↑	[22,74]
	fat body	↑	[22]

↑ increase, ↓ decrease, * difference between castes.

**Table 3:** General tendencies of aspartate transaminase activities in hemolymph of honeybees under the influence of various factors.

Factor	Tissue	Trend	Literature
**Age**	hemolymph	↑	[34,111]
***Varroa destructor***	hemolymph	↓	[116]
**E-field at frequency 50 Hz**	hemolymph	↓	[117]
**E-field at frequency 900 MHz**	hemolymph	~	[106]
**Exposure to common tansy extract (a natural substitute for synthetic pesticides)**	hemolymph	↑	[70]
**CBD extract**	hemolymph	↑	[34]
**Treatment with bromfenvinphos**	hemolymph	↓	[73]
**Treatment with amphotericin-B.**	hemolymph	↓	[17]
**Consumed curcumin**	hemolymph	↑	[35]
**Consumed caffeine**	hemolymph	↑	[36]
**Consumed coenzyme Q10**	hemolymph	↑	[109]
**Imidacloprid**	hemolymph	↓	[15]

↑ increase, ↓ decrease, ~ not much difference.

**Table 4:** General tendencies of alanine transaminase activities in hemolymph of honeybees under the influence of various factors.

Factor	Tissue	Trend	Literature
**Age**	hemolymph	↑	[34,111]
***Varroa destructor***	hemolymph	↓	[116]
**E-field at frequency 50 Hz**	hemolymph	↓	[117]
**E-field at frequency 900 MHz**	hemolymph	~	[106]
**Treatment with bromfenvinphos**	hemolymph	↓	[73]
**Treatment with amphotericin-B.**	hemolymph	↓	[17]
**CBD extract**	hemolymph	↑	[34]
**Consumed curcumin**	hemolymph	↑	[35]
**Consumed caffeine**	hemolymph	↑	[36]
**Consumed coenzyme Q10**	hemolymph	↑	[109]
**Imidacloprid**	hemolymph	↓	[15]

↑ increase, ↓ decrease, ~ not much difference.

**Table 5:** General tendencies of alkaline phosphatase activities in hemolymph of honeybees under the influence of various factors.

Factor	Tissue	Trend	Literature
**Age**	hemolymph	↑	[111]
***Varroa destructor***	hemolymph	↓	[116]
**E-field at frequency 50 Hz**	hemolymph	↓	[117]
**E-field at frequency 900 MHz**	hemolymph	~	[106]
**Treatment with amphotericin-B.**	hemolymph	~	[17]
**Treatment with bromfenvinphos**	hemolymph	↓	[73]
**CBD extract**	hemolymph	↑	[34]
**Consumed curcumin**	hemolymph	↑	[35]
**Consumed caffeine**	hemolymph	↑	[36]
**Consumed coenzyme Q10**	hemolymph	↑	[109]
**Imidacloprid**	hemolymph	↓	[15]

↑ increase, ↓ decrease, ~ not much difference.

**Table 6:** General tendencies of antioxidant enzyme activities in different tissues of honeybees under the influence of various factors.

Factor	Enzymes	Tissue	Trend	Literature
**Age**	CAT	fat body,	↑	[27,128]
		homogenates from entire abdomens	↑	[129,130]
		hemolymph	↑	[33]
	SOD	fat body,	↓	[128]
		hemolymph	↑	[27,33]
***Varroa*** ***destructor***	CAT	hemolymph, fat body	↓	[27]
	SOD, GST	hemolymph, fat body	↑	
**E-field at frequency 50 Hz**	CAT, SOD,	hemolymph	↑	[124]
***Nosema* spp.**	CAT, SOD, GST	homogenates from entire abdomens	↑	[48]
**Consumed curcumin**	CAT, SOD, GST, GPx,	hemolymph	↑	[35]
**Consumed caffeine**	SOD, CAT, GST, GPx,	hemolymph	↑	[36]
**Treatment with bromfenvinphos**	SOD, CAT, GST, GPx	hemolymph	↓	[73]
**Oxalic acid treatment**	CAT, GST	hemolymph	~	[49]
**Consumed coenzyme Q10**	SOD, CAT, GST, GPx	hemolymph	↑	[109]
**Adulteration of the wax foundation**	CAT, SOD, GST, GPx	hemolymph	↓	[19]
**Various monofloral diets**	SOD, GST, GPx	hemolymph, fat body	↑	[130]
**Urban and rural areas**	GST	brain, fat body	~	[119]

↑ increase, ↓ decrease, ~ not much difference.

**Table 7:** General tendencies of triacylglycerol concentrations in hemolymph and fat body of honeybees under the influence of various factors.

Factor	Tissue	Trend	Literature
**Age**	hemolymph	↓	[34]
**E-field at frequency 50 Hz**	hemolymph	↓	[65]
**Treatment with amphotericin-B.**	hemolymph	↑	[17]
***Varroa destructor***	hemolymph	↓	[107]
***Nosema* spp.**	hemolymph	~	[67]
**CBD extract**	hemolymph	↑	[34]
**Consumed curcumin**	hemolymph	↑	[35]
**Various monofloral diets**	hemolymph, fat body	↑	[22]

↑ increase, ↓ decrease, ~ not much difference.

## Data Availability

Data are contained within the article.
